# Comparative Screening of Digestion Tract Toxic Genes in *Proteus mirabilis*

**DOI:** 10.1371/journal.pone.0151873

**Published:** 2016-03-24

**Authors:** Xiaolu Shi, Yiman Lin, Yaqun Qiu, Yinghui Li, Min Jiang, Qiongcheng Chen, Yixiang Jiang, Jianhui Yuan, Hong Cao, Qinghua Hu, Shenghe Huang

**Affiliations:** 1 School of Public Health and Tropical Medicine, Southern Medical University, Guangzhou, 510515, China; 2 Shenzhen Center for Disease Control and Prevention, Shenzhen, China; Jilin University, CHINA

## Abstract

*Proteus mirabilis* is a common urinary tract pathogen, and may induce various inflammation symptoms. Its notorious ability to resist multiple antibiotics and to form urinary tract stones makes its treatment a long and painful process, which is further challenged by the frequent horizontal gene transferring events in *P*. *mirabilis* genomes. Three strains of *P*. *mirabilis* C02011/C04010/C04013 were isolated from a local outbreak of a food poisoning event in Shenzhen, China. Our hypothesis is that new genes may have been acquired horizontally to exert the digestion tract infection and toxicity. The functional characterization of these three genomes shows that each of them independently acquired dozens of virulent genes horizontally from the other microbial genomes. The representative strain C02011 induces the symptoms of both vomit and diarrhea, and has recently acquired a complete type IV secretion system and digestion tract toxic genes from the other bacteria.

## Introduction

The gram-negative anaerobic bacterium *Proteus mirabilis* is one of the major *Proteus* infectious factors, and may cause severe pain by forming stones in the urinary tract [1]. *P*. *mirabilis* may induce various pathogenic symptoms, including fever, chills and chest pain, etc [2]. Its motility and adhesion to solid surface makes *P*. *mirabilis* extremely easy to propagate through medical devices in hospitals [2]. *P*. *mirabilis* is also notorious for its ability to actively acquire antibiotic resistance and infectious toxin genes from other microbial genomes through horizontal gene transferring [3, 4].

Quite a number of molecular typing technologies have been developed for a prompt screening of *Proteus mirabilis* in clinical samples. The protein UreC is the alpha subunit of the urea degradation pathway encoded by *P*. *mirabilis* [5]. The second metabolite repressor gene *rsmA* is involved in the swarming motility and the expression regulation of virulence factors in *P*. *mirabilis* [6]. *P*. *mirabilis* also encodes a kidney damage inducer, the hemolysin HpmA, which shows significant cytotoxicity [7]. The virulence factor metalloprotease ZapA is demonstrated to regulate the IgA hydrolysis process by the inhibiting profiles [8]. A number of social network analyzing techniques were applied to characterize the disease-associated microRNAs [9, 10], and the improved detection performance suggests that techniques from different areas may be complementary to each other for the challenging detection problem for the disease-microRNA association [11, 12], which may benefit the detection problem of pathogenicity genes in *P*. *mirabilis*.

This study hypothesized that *P*. *mirabilis* exerts its infectious and toxic functions in the new location, digestion tract, through site specific genes. Three *P*. *mirabilis* strains isolated from a local outbreak of food poison, and two more non-toxic strains were isolated from the same physical location after the outbreak event. The genes and repetitive elements were annotated for all the *P*. *mirabilis* strains. A comprehensive screening of the digestion tract toxicity (DTT) specific genes is carried out for the *P*. *mirabilis* infectious strains. The comparative study supports our hypothesis that some strain specific genes may serve as candidates for rapid strain typing and treatment selection.

## Material and Methods

### Strain isolations

During an outbreak of a food-poisoning event in Shenzhen in June of 2002, three strains of *P*. *mirabilis* were collected from samples of different patients. The strain C02011 was isolated from the vomit sample of a patient, and it has the same Pulsed-Field Gel Electrophoresis (PFGE) band of the *P*. *mirabilis* strain isolated from the diarrhea stool. So the strain C02011 is kept as a representative strain for further analysis. The strain C04010 was isolated from the diarrhea stool sample of a patient in the same event. Another strain C04013 was isolated from the food taken by the patients immediately before this food-poisoning event.

These three strains of *P*. *mirabilis* were compared with two locally isolated strains and two reference strains. Another two strains of *P*. *mirabilis* C02034 and B02005 were isolated from the food and food worker’s stool during non-outbreak time at the same physical location. The two reference strains HI4320 [2] and BB2000 [13] were urinary tract infection strains, and were also chosen as the control samples for the comparative genomics investigation.

This study was approved by the Ethics Committee of Shenzhen Center for Disease Control and Prevention on June 9, 2002. The authors did not collect the samples directly from the patients, and received the samples from the local hospitals. This study investigated the genome sequencing data of the pathogens and did not access the patients’ personal data. So no informed consent forms were collected from the patients.

### Genomic sequencing and gene annotation

The five newly isolated strains were sequenced and assembled by the BGI-Shenzhen. Two insert fragment lengths 500 and 2000 bps were chosen for the paired-end (PE) sequencing using the Illumina HiSeq-2000 machine. The read lengths were set as PE-90 and PE-49 for the 500-bp and 2000-bp libraries, respectively. The 500-bp paired-end reads were *de novo* assembled using the program SOAPdenovo version 1.06, and the contigs were further connected into scaffolds using both 500-bp and 2000-bp PE reads [14]. The genes were annotated using the program Glimmer version 3.02 [15]. The functions of each gene were annotated using the NCBI BLAST based on the databases NCBI NR [16], SwissProt/Trembl [17], COG [18] and KEGG [19], respectively. The data are summarized in the Results and discussion section. Transcriptome is not investigated in this study and the microRNA precursor genes will be characterized by both *in silico* techniques [20] and RNA-seq experiments [21].

### Annotation of repetitive elements

Repetitive elements are not annotated by default for a genome sequencing service, and a comprehensive annotation is conducted for the five sequenced strains and the two reference strains of *P*. *mirabilis* investigated in this study.

Repetitive elements were annotated by the RepeatMasker version 4.05 [22]. The tandem repeats were screened by the *de novo* program TRF version 4.04 [23]. Prokaryotic genomes have transposable elements like Insertion Sequence (IS) elements [24, 25] and Miniature Inverted-repeat Transposable Elements (MITEs) [26–28]. Full copies of IS elements were detected and curated by multiple sequence alignments of IS encoded transposase genes, using the program MEGA version 6.06 [29]. Full copies of detected IS elements and all other full-length IS copies from the database ISfinder [30] were used as templates to screen for all the IS copies in the seven strains of *P*. *mirabilis*. No MITEs were detected by the program MUST version 1.0 [31].

CRISPRs are a series of consecutive short repeats with spacers in between, and the spacer sequences are usually acquired from the invasive foreign elements [32, 33]. The program CRISPRfinder [34] was used to detect CRISPRs in the seven investigated *P*. *mirabilis* genomes.

### Generation of the phylogenetic tree

A phylogenetic tree based on the 16S ribosomal RNA (rRNA) genes is generated for the five *P*. *mirabilis* strains sequenced by this study and the three additional *P*. *mirabilis* reference strains published elsewhere. The 16S rRNA genes of the five newly sequenced *P*. *mirabilis* strains are annotated as described in the above sections. The 16S rRNA genes of the two *P*. *mirabilis* strains HI4320 and BB2000 are collected from the NCBI database. C05028 is another publicly available strain with DTT function [35], and its 16S rRNA gene is extracted using NCBI MegaBlast version 2.2.32+ [36] with the 16S rRNA of *P*. *mirabilis* HI4320 as template. The phylogenetic tree is rooted at *Proteus penneri* ATCC 35198, whose 16S rRNA gene is detected with the template gene from the strain HI4320 using the program NCBI BLASTN version 2.2.32+ [36]. The *E-value* 0.0 supports that the two microbial genomes are close relatives with enough statistical significance. The phylogenetic tree is generated using Phylogeny.fr [37]. All the programs are executed using the default parameters.

### Screening for the strain specific genes

A highly sensitive screening of whether a gene has a homolog in a given genome is conducted using the NCBI MegaBLAST version 2.2.32+ [36] with the default parameters. Only those genes with no homologs are kept as strain specific candidates.

## Results and Discussion

### *In vitro* PFGE strain typing

Three isolated *P*. *mirabilis* strains demonstrate an abnormal infectious ability and toxicity to the digestion tract, but two other strains don’t have such ability. So the first question is whether the existing molecular typing technologies may differentiate these strains by their pathogenesis.

PFGE is a widely used rapid subtyping technique for the macrorestriction analysis of pathogens, as recommended by the PulseNet laboratories [38]. 50 U of the restriction enzyme SfiI in the 200 ml buffer was used to generate the genomic fragment profile, and a standard protocol was followed [39]. After the gel was scanned as the TIFF format image, a dendrogram tree was calculated using the software BioNumerics version 5.0 (Applied Maths BVBA, Belgium), as similar in [40].

**Fig 1:**
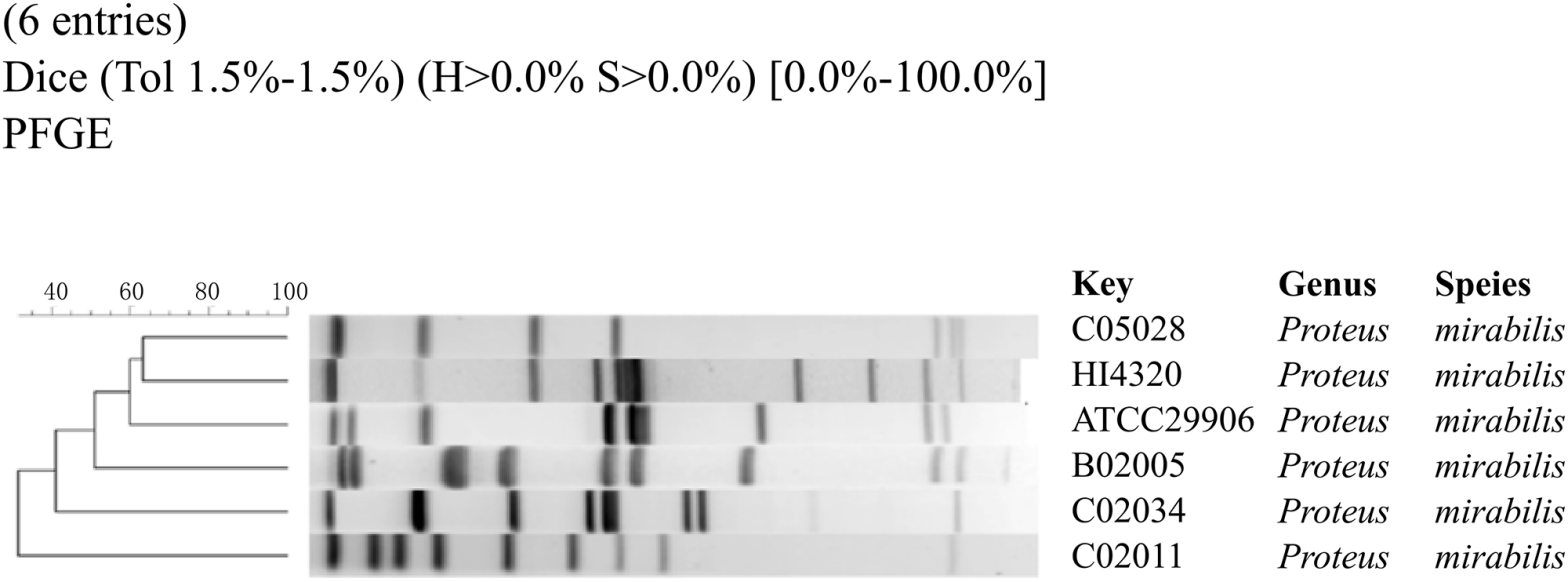
The dendrogram tree of six *P*. *mirabilis* strains. Strains C05028 and C02011 are digestion tract infectious. Strains HI4320 and ATCC29906 are urinary tract infectious. Strains B02005 and C02034 are non-infectious strains.

The restriction enzyme SfiI cuts the genomic sequence into fragments of lengths between 20–700 kbps, and the PFGE profiles in Fig 1 shows significant differences even between closely related *P*. *mirabilis* strains. The inconsistency of PFGE profiles of these six *P*. *mirabilis* strains with their pathogenicity suggests that these genomes may under strong selection pressures for genomic changes, and may undergo both intra-genome structural variations and inter-genome/strain horizontal transferring.

### *In vitro* PCR strain typing

The polymerase chain reaction (PCR) is also a common technique to subtype pathogens based on the existences of known pathogenic factors [41]. The PCR-based screening of the genes *ureC*, *rsmA*, *hpmA* and *zapA* were conducted in the five newly isolated strains of *P*. *mirabilis*. As shown in Table 1, the primers for these four genes were designed in-house, and a standard PCR protocol was followed.

**Table 1:** PCR primers for the four genes.

Target	pName	Primer (5’-3’)	Product (bps)
*ureC*	*ureC*-F	GTATTCGTCGCGAAACCATT	553
	*ureC*-R	ATATGACGACAGCCCTCCAC	
*rsmA*	*rsmA*-F	TAGCGAGTGTTGACGAGTGG	562
	*rsmA*-R	AGCGAGGTGAAGAACGAGAA	
*hpmA*	*hpmA*-F	CCAGTGAATTAACGGCAGGT	654
	*hpmA*-R	CGTGCCCAGTAATGGCTAAT	
*zapA*	*zapA*-F	AACGCAGGTCAGAATGTTCC	553
	*zapA*-R	TATCTTGTCCACGACCACCA	

Column pName lists the names of the primers.

Unfortunately, there is no difference between the digestion tract toxic strains and the other strains, since all the five strains encode all these four genes, as shown in Table 2 and S1 Fig. So the existing pathogen subtyping techniques are not precise enough to differentiate these five *P*. *mirabilis* strains.

**Table 2:** Currently adopted molecular typing technologies of biomarker genes in the infectious bacterial strains.

Strain	Detail	*ureC*	*rsmA*	hemolysin (*hpmA*)	metalloprotease (*zapA*)
C02011	Patient vomit	+	+	+	+
C04010	Patient stool	+	+	+	+
C04013	Poison food	+	+	+	+
C02034	Normal food	+	+	+	+
B02005	Normal stool	+	+	+	+

The first three strains C02011, C04010 and C04013 induce DTT function, while the other two strains don’t.

This study carried out a comprehensive screening of functional elements that are specific to these three digestion tract toxic strains, compared against the other two digestion tract infectious strains. These strain specific genes may serve as candidate subtyping targets.

### Summary of the seven *P*. *mirabilis* strains

*P*. *mirabilis* strain C02011 induces the symptoms of both vomit and diarrhea, and is chosen as the reference genome for the screening of DTT genes. Among the seven *P*. *mirabilis* strains, C02034 and HI4320 have the genome sizes larger than 4 Mbps, and all the other strains have a genome size between 3.80 and 3.85 Mbps, as shown in Table 3. It’s interesting to observe that these two largest genomes also have the largest numbers of IS elements, with 28 and 24 in C02034 and HI4320, respectively. This supports the observation that IS number tends to be positively correlated with the genome size [25, 42]. All the seven strains have a similar GC content between 38.38–38.88%. The Pearson’s test generates the *P-value* = 7.341e-4 for the correlation between the Gsize and Gene# for the seven strains of *P*. *mirabilis*. So a large genome tends to encode more genes. Only the two strains C02011 and C02034 have 2 CRISPRs, respectively.

**Table 3:** Annotation summary of the seven *P*. *mirabilis* strains investigated in this study.

Strain	Gsize (bps)	GC (%)	Gene#	IS	CRISPR	rDrug	Toxin	T4SS	DTT specific
C02011	3802255	38.45	3,463	20	2	45	17	14	62
C04010	3803688	38.38	3,465	17	0	46	30	3	66
C04013	3844051	38.46	3,511	11	0	46	10	3	59
B02005	3830296	38.56	3,516	12	0	45	0	3	-
C02034	4166171	38.62	3,761	28	2	51	0	3	-
BB2000	3846754	38.60	3,455	15	0	42	-	3	-
HI4320	4063606	38.88	3,607	24	0	41	-	3	-

Columns “Gsize (bps)” and “GC” give the estimated genome size and the G+C content of each strain. The numbers of annotated genes and IS elements are given in the columns “Gene#” and “IS”. The annotated CRISPR number is given in the column “CRISPR”. The numbers of drug resistant genes and toxin genes are based on the gene function annotation and listed in the columns “rDrug” and “Toxin”, respectively. The gene number in the T4SS is in the column “T4SS”, which is summarized from the gene annotations. The column “DTT specific” gives the number of genes with no homologs in the non-toxin *P*. *mirabilis* strains. “-”means that this item is not analyzed for this strain. The gene annotation of the strain C05028 is not publicly available, and is not included in this table.

All the seven *P*. *mirabilis* strains encode drug resistance genes, but only the three strains with DTT function encode toxin genes, as shown in Table 3. The type IV secretion system (T4SS) does not seem to be a major secretion system in *P*. *mirabilis*, with 3 T4SS genes in each strain except for C02011. And each of the three digestion tract toxic strains encodes ~60 genes, which are not detected in the other *P*. *mirabilis* strains.

This study will focus on the DTT specific genes that may facilitate the radical pathogenesis during the local food poison event.

### Phylogenetic traces

*P*. *mirabilis* is notorious for its infection and stone forming in the urinary tract, and usually causes severe pain. The two publicly available strains BB2000 and HI4320 may induce various whole body symptoms when infecting the urinary tract [2, 13]. But the five strains isolated from Shenzhen and the strain C05028 seem to have gained the ability to infect the digestion tract, and four of them demonstrate DTT function during their infections.

**Fig 2:**
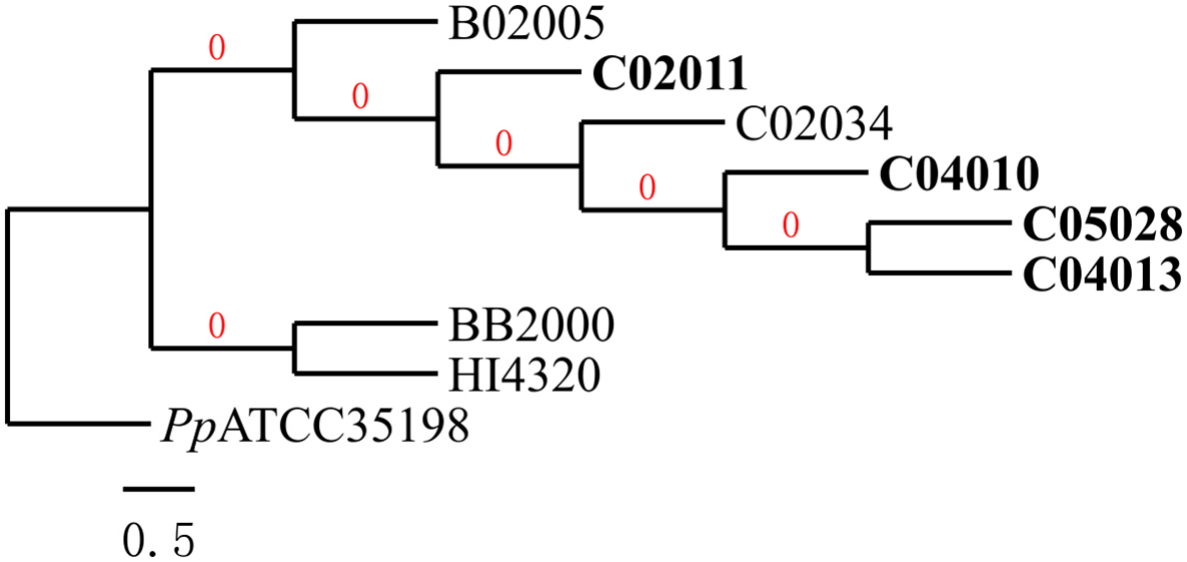
The phylogenetic tree of the seven *P*. *mirabilis* strains based on the 16S rRNA genes. The Cladogram tree is rooted at *Proteus penneri* ATCC 35198, and generated by the Phylogeny.fr. The three *P*. *mirabilis* strains inducing DTT function are highlighted in the bold font.

The five locally isolated strains of *P*. *mirabilis* together with the strain C05028 originate from the same common ancestor, whereas the other two urinary tract infectious strains BB2000 and HI4320 originate from another common ancestor, based on the phylogenetic tree in Fig 2. The two non-toxic strains C02034 and B02005 do not show a clear separation from the three digestion tract toxic (DTT) strains on the phylogenetic tree. This suggests that genes specific to DTT strains may play an essential role in infecting and inducing toxicity to the digestion tract.

### DTT specific elements

**Fig 3:**
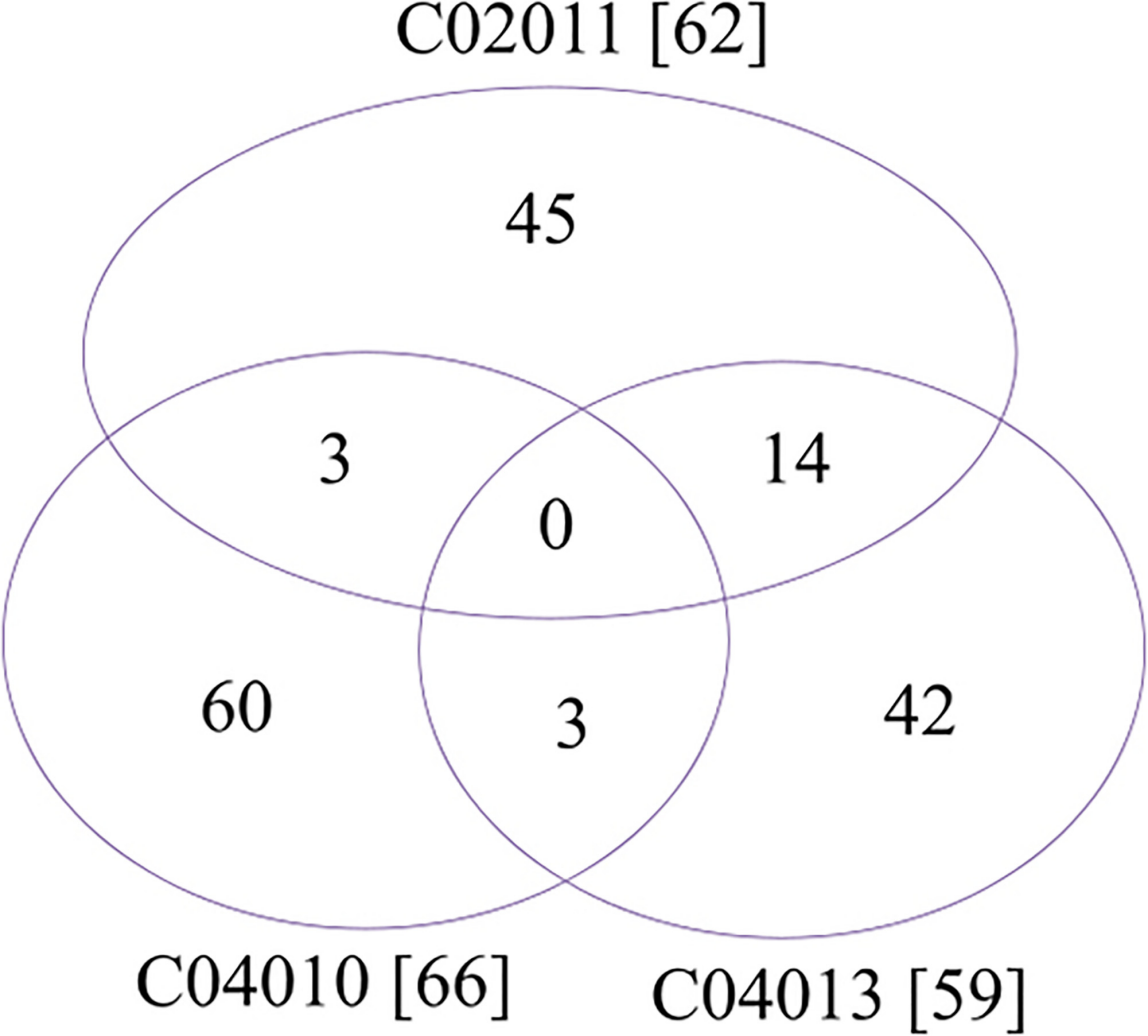
Venn plot of the strain specific genes in the three *P*. *mirabilis* DTT strains. The numbers in the brackets are the numbers of DTT specific genes compared against the other *P*. *mirabilis* strains.

Most of the DTT specific genes are also strain specific, as shown in Fig 3. The DTT specific genes have no homologs in the two non-toxic strains C02034 and B02005 and the two urinary tract infectious strains HI4320 and BB2000, and over 70% of them are only detected in one of the three DTT strains. And all 45 genes specific in the strain C02011 does not have detectable homologs in the publicly available strain *P*. *mirabilis* C05028. By considering the phylogenetic relationships among the three DTT strains (as in Fig 2), the current data strongly suggests that all the three *P*. *mirabilis* DTT strains are actively and independently acquiring foreign genes from the environmental neighboring microbes.

### Core module for DTT function

The most infectious and toxic strain C02011 encodes 45 genes with no homologs in all the seven other strains. This suggests that these genes may have been acquired through the mechanism of horizontal gene transferring. The function annotation shows that these 45 genes do not introduce new antibiotic resisting abilities into the strain C02011, as shown in Table 4 and S1 Table.

**Table 4:** 45 genes specific in *P*. *mirabilis* C02011.

Scaffold	Start	End	Strand	gene_id	Annotated protein
fa_scaffold3	465840	465962	+	**C02011GL001038**	
fa_scaffold3	465952	466815	+	**C02011GL001039**	
fa_scaffold3	466833	467849	+	**C02011GL001040**	Putative phage gene
fa_scaffold3	467846	468379	+	**C02011GL001041**	
fa_scaffold3	468467	468952	+	**C02011GL001042**	Putative phage gene
fa_scaffold3	468949	469179	+	**C02011GL001043**	
fa_scaffold3	469224	471878	+	**C02011GL001044**	Putative inner membrane protein
fa_scaffold3	472166	472393	+	**C02011GL001045**	Putative phage gene
fa_scaffold3	472383	472538	+	**C02011GL001046**	
fa_scaffold3	473119	473679	+	**C02011GL001047**	
fa_scaffold3	473682	475016	+	**C02011GL001048**	Retro-type reverse transcriptase
fa_scaffold3	1068445	1069563	-	C02011GL001596	
fa_scaffold3	1069568	1070161	-	C02011GL001597	
fa_scaffold3	1535544	1536122	-	C02011GL002036	
fa_scaffold3	1536545	1537150	+	C02011GL002037	Integrase
fa_scaffold3	1538064	1538282	+	C02011GL002039	
fa_scaffold3	1538847	1539260	-	C02011GL002041	
fa_scaffold3	1543281	1543748	-	C02011GL002049	
fa_scaffold3	1543975	1544586	-	C02011GL002050	
fa_scaffold3	1544614	1545003	-	C02011GL002051	
fa_scaffold3	1809941	1810177	-	**C02011GL002280**	
fa_scaffold3	1810202	1810420	-	**C02011GL002281**	
fa_scaffold3	1810632	1811384	-	**C02011GL002282**	Putative phage regulatory protein
fa_scaffold3	1811504	1812076	-	**C02011GL002283**	BRO family protein
fa_scaffold3	1812403	1813122	+	**C02011GL002284**	
fa_scaffold3	1823562	1824128	-	C02011GL002300	Putative phage terminase
fa_scaffold3	1824196	1824423	+	C02011GL002301	
fa_scaffold3	1824634	1824879	-	C02011GL002302	
fa_scaffold3	1825050	1825649	-	C02011GL002303	KilA-N domain-containing protein
fa_scaffold6	547377	548129	-	**C02011GL003316**	MobC
fa_scaffold6	548138	549889	-	**C02011GL003317**	MobB
fa_scaffold6	549859	550503	-	**C02011GL003318**	
fa_scaffold6	550993	551298	-	**C02011GL003319**	ORF15
fa_scaffold6	551334	551708	-	**C02011GL003320**	
fa_scaffold6	551729	552754	-	**C02011GL003321**	T4SS virB11
fa_scaffold6	552744	554012	-	**C02011GL003322**	T4SS virB10
fa_scaffold6	554056	554961	-	**C02011GL003323**	T4SS virB9
fa_scaffold6	554961	555644	-	**C02011GL003324**	T4SS virB8
fa_scaffold6	555866	556936	-	**C02011GL003325**	T4SS virB6
fa_scaffold6	557184	557885	-	**C02011GL003326**	T4SS virB5
fa_scaffold6	557903	560641	-	**C02011GL003327**	T4SS virB4
fa_scaffold6	560654	560833	-	**C02011GL003328**	T4SS virB2
fa_scaffold6	560947	561699	-	**C02011GL003329**	T4SS virB1
fa_scaffold6	562549	563136	-	**C02011GL003330**	
fa_scaffold6	590486	591754	-	C02011GL003343	Integrase

The three large genomic islands are highlighted by a bold font in the column “gene_id”. The gene annotations are summarized from the S1 Table.

Three large genomic islands with at least 5 consecutively distributed genes are detected in the strain C02011. The largest genomic island encodes 15 genes, including a complete T4SS. The transmembrane secretion assembly T4SS consists of multiple component proteins, and is utilized by various gram-negative bacteria to transport virulent factors between the pathogen and the host cells [43]. Among the sequenced *P*. *mirabilis* strains, T4SS is only observed in the urinary tract infecting strain HI4320 [2, 44, 45]. And the T4SS in the strain C02011 does not show any similarity on the primary sequence level to that in HI4320, which supports the observation of little sequence conservation of T4SS modules from different bacteria [43, 46–49]. This genomic island also encodes three genes with significant homologs in the plasmid pMET-1 from the Gammaproteobacterium *Klebsiella pneumoniae*[50]. Among the three genes, *mobB* encodes three transmembrane domains and ORF15 has a 5’ signal peptide.

Another two genomic islands encode genes with various phage functionalities, as shown in the S1 Table.

Besides these transmembrane transporting genes, the strain C02011 also encodes a gene C02011GL002300 homologous to the phage terminase from a food poison pathogen *Yersinia enterocolitica* 8081, as shown in the S1 Table. *Y*. *enterocolitica* 8081 belongs to the serotype 0:8 and biotype 1B of *Y*. *enterocolitica*, and may induce severe diarrhea [51]. The most recent common ancestor between *P*. *mirabilis* and *Y*. *enterocolitica* on the phylogenetic tree is Enterobacteriaceae on the family level. The data suggests that *P*. *mirabilis* C02011 may have horizontally acquired severe digestion tract toxic genes from another genus *Yersinia*.

## Supporting Information

S1 Fig PCR products after gel electrophoresis of the four genes in the five investigated strains of P. mirabilis. (a) ureC, (b) rsmA, (c) hpmA, and (d) zapA. The lanes M and C are the 100-bp ladder and the control ddH2O lanes, respectively. The lanes 1 through 5 are the P. mirabilis strains C02011, C04010, C04013, C02034 and B02005, respectively.
(DOCX)

S1 Table Annotations of the 45 genes specific in P. mirabilis C02011. The three candidate genomic islands are highlighted by bold font in the column “gene_id”. The annotation is provided by the BGI, using the reference databases NCBI NR, SwissProt, Trembl, COG and KEGG.
(DOCX)

## References

[1] Li X, Zhao H, Lockatell CV, Drachenberg CB, Johnson DE, Mobley HL. Visualization of Proteus mirabilis within the matrix of urease-induced bladder stones during experimental urinary tract infection. Infection and immunity. 2002;70(1):389–94. 11748205 10.1128/IAI.70.1.389-394.2002 PMC127628

[2] Pearson MM, Sebaihia M, Churcher C, Quail MA, Seshasayee AS, Luscombe NM, et al Complete genome sequence of uropathogenic Proteus mirabilis, a master of both adherence and motility. Journal of bacteriology. 2008;190(11):4027–37. 10.1128/JB.01981-07 18375554 PMC2395036

[3] Leverstein-van Hall MA, MB HE, TD AR, Paauw A, Fluit AC, Verhoef J. Multidrug resistance among Enterobacteriaceae is strongly associated with the presence of integrons and is independent of species or isolate origin. The Journal of infectious diseases. 2003;187(2):251–9. 10.1086/345880 . 12552449

[4] Pagani L, Migliavacca R, Pallecchi L, Matti C, Giacobone E, Amicosante G, et al Emerging extended-spectrum beta-lactamases in Proteus mirabilis. Journal of clinical microbiology. 2002;40(4):1549–52. 11923394 10.1128/JCM.40.4.1549-1552.2002 PMC140357

[5] Sriwanthana B, Mobley HL. Proteus mirabilis urease: histidine 320 of UreC is essential for urea hydrolysis and nickel ion binding within the native enzyme. Infection and immunity. 1993;61(6):2570–7. 8500894 10.1128/iai.61.6.2570-2577.1993 PMC280886

[6] Liaw SJ, Lai HC, Ho SW, Luh KT, Wang WB. Role of RsmA in the regulation of swarming motility and virulence factor expression in Proteus mirabilis. Journal of medical microbiology. 2003;52(Pt 1):19–28. 10.1099/jmm.0.05024-0 . 12488561

[7] Cestari SE, Ludovico MS, Martins FH, da Rocha SP, Elias WP, Pelayo JS. Molecular detection of HpmA and HlyA hemolysin of uropathogenic Proteus mirabilis. Current microbiology. 2013;67(6):703–7. 10.1007/s00284-013-0423-5 . 23884594

[8] Carson L, Cathcart GR, Scott CJ, Hollenberg MD, Walker B, Ceri H, et al Comprehensive inhibitor profiling of the Proteus mirabilis metalloprotease virulence factor ZapA (mirabilysin). Biochimie. 2011;93(10):1824–7. 10.1016/j.biochi.2011.06.030 . 21762758

[9] Zou Q, Li J, Wang C, Zeng X. Approaches for recognizing disease genes based on network. BioMed research international. 2014;2014:416323 10.1155/2014/416323 24707485 PMC3953674

[10] Zou Q, Li J, Hong Q, Lin Z, Wu Y, Shi H, et al Prediction of MicroRNA-Disease Associations Based on Social Network Analysis Methods. BioMed research international. 2015;2015:810514 10.1155/2015/810514 26273645 PMC4529919

[11] Zeng X, Zhang X, Zou Q. Integrative approaches for predicting microRNA function and prioritizing disease-related microRNA using biological interaction networks. Briefings in bioinformatics. 2015 10.1093/bib/bbv033 . 26059461

[12] Zou Q, Li J, Song L, Zeng X, Wang G. Similarity computation strategies in the microRNA-disease network: a survey. Briefings in functional genomics. 2016;15(1):55–64. 10.1093/bfgp/elv024 . 26134276

[13] Sullivan NL, Septer AN, Fields AT, Wenren LM, Gibbs KA. The Complete Genome Sequence of Proteus mirabilis Strain BB2000 Reveals Differences from the P. mirabilis Reference Strain. Genome announcements. 2013;1(5). 10.1128/genomeA.00024-13 24009111 PMC3764406

[14] Luo R, Liu B, Xie Y, Li Z, Huang W, Yuan J, et al SOAPdenovo2: an empirically improved memory-efficient short-read de novo assembler. GigaScience. 2012;1(1):18 10.1186/2047-217X-1-18 23587118 PMC3626529

[15] Delcher AL, Bratke KA, Powers EC, Salzberg SL. Identifying bacterial genes and endosymbiont DNA with Glimmer. Bioinformatics. 2007;23(6):673–9. 10.1093/bioinformatics/btm009 17237039 PMC2387122

[16] O'Leary NA, Wright MW, Brister JR, Ciufo S, Haddad D, McVeigh R, et al Reference sequence (RefSeq) database at NCBI: current status, taxonomic expansion, and functional annotation. Nucleic acids research. 2016;44(D1):D733–45. 10.1093/nar/gkv1189 26553804 PMC4702849

[17] McMillan LE, Martin AC. Automatically extracting functionally equivalent proteins from SwissProt. BMC bioinformatics. 2008;9:418 10.1186/1471-2105-9-418 18838004 PMC2576269

[18] Tatusov RL, Galperin MY, Natale DA, Koonin EV. The COG database: a tool for genome-scale analysis of protein functions and evolution. Nucleic acids research. 2000;28(1):33–6. 10592175 10.1093/nar/28.1.33 PMC102395

[19] Kanehisa M, Sato Y, Kawashima M, Furumichi M, Tanabe M. KEGG as a reference resource for gene and protein annotation. Nucleic acids research. 2016;44(D1):D457–62. 10.1093/nar/gkv1070 26476454 PMC4702792

[20] Wei L, Liao M, Gao Y, Ji R, He Z, Zou Q. Improved and Promising Identification of Human MicroRNAs by Incorporating a High-Quality Negative Set. IEEE/ACM transactions on computational biology and bioinformatics / IEEE, ACM. 2014;11(1):192–201. 10.1109/TCBB.2013.146 . 26355518

[21] McGettigan PA. Transcriptomics in the RNA-seq era. Current opinion in chemical biology. 2013;17(1):4–11. 10.1016/j.cbpa.2012.12.008 . 23290152

[22] Tempel S. Using and understanding RepeatMasker. Methods in molecular biology. 2012;859:29–51. 10.1007/978-1-61779-603-6_2 . 22367864

[23] Benson G. Tandem repeats finder: a program to analyze DNA sequences. Nucleic acids research. 1999;27(2):573–80. 9862982 10.1093/nar/27.2.573 PMC148217

[24] Siguier P, Gourbeyre E, Chandler M. Bacterial insertion sequences: their genomic impact and diversity. FEMS microbiology reviews. 2014;38(5):865–91. 10.1111/1574-6976.12067 . 24499397 PMC7190074

[25] Zhou F, Olman V, Xu Y. Insertion Sequences show diverse recent activities in Cyanobacteria and Archaea. BMC genomics. 2008;9:36 10.1186/1471-2164-9-36 18218090 PMC2246112

[26] Chen Y, Zhou F, Li G, Xu Y. A recently active miniature inverted-repeat transposable element, Chunjie, inserted into an operon without disturbing the operon structure in Geobacter uraniireducens Rf4. Genetics. 2008;179(4):2291–7. 10.1534/genetics.108.089995 18660544 PMC2516098

[27] Zhou F, Tran T, Xu Y. Nezha, a novel active miniature inverted-repeat transposable element in cyanobacteria. Biochemical and biophysical research communications. 2008;365(4):790–4. 10.1016/j.bbrc.2007.11.038 . 18035045

[28] Brugger K, Redder P, She Q, Confalonieri F, Zivanovic Y, Garrett RA. Mobile elements in archaeal genomes. FEMS microbiology letters. 2002;206(2):131–41. . 11814653 10.1111/j.1574-6968.2002.tb10999.x

[29] Tamura K, Stecher G, Peterson D, Filipski A, Kumar S. MEGA6: Molecular Evolutionary Genetics Analysis version 6.0. Molecular biology and evolution. 2013;30(12):2725–9. 10.1093/molbev/mst197 24132122 PMC3840312

[30] Siguier P, Perochon J, Lestrade L, Mahillon J, Chandler M. ISfinder: the reference centre for bacterial insertion sequences. Nucleic acids research. 2006;34(Database issue):D32–6. 10.1093/nar/gkj014 16381877 PMC1347377

[31] Chen Y, Zhou F, Li G, Xu Y. MUST: a system for identification of miniature inverted-repeat transposable elements and applications to Anabaena variabilis and Haloquadratum walsbyi. Gene. 2009;436(1–2):1–7. 10.1016/j.gene.2009.01.019 . 19393167

[32] Sorek R, Kunin V, Hugenholtz P. CRISPR—a widespread system that provides acquired resistance against phages in bacteria and archaea. Nature reviews Microbiology. 2008;6(3):181–6. 10.1038/nrmicro1793 . 18157154

[33] Deveau H, Garneau JE, Moineau S. CRISPR/Cas system and its role in phage-bacteria interactions. Annual review of microbiology. 2010;64:475–93. 10.1146/annurev.micro.112408.134123 . 20528693

[34] Grissa I, Vergnaud G, Pourcel C. CRISPRFinder: a web tool to identify clustered regularly interspaced short palindromic repeats. Nucleic acids research. 2007;35(Web Server issue):W52–7. 10.1093/nar/gkm360 17537822 PMC1933234

[35] Shi X, Zhu Y, Li Y, Jiang M, Lin Y, Qiu Y, et al Genome Sequence of Proteus mirabilis Clinical Isolate C05028. Genome announcements. 2014;2(2). 10.1128/genomeA.00167-14 24675851 PMC3968329

[36] Zhang Z, Schwartz S, Wagner L, Miller W. A greedy algorithm for aligning DNA sequences. Journal of computational biology: a journal of computational molecular cell biology. 2000;7(1–2):203–14. 10.1089/10665270050081478 . 10890397

[37] Dereeper A, Guignon V, Blanc G, Audic S, Buffet S, Chevenet F, et al Phylogeny.fr: robust phylogenetic analysis for the non-specialist. Nucleic acids research. 2008;36(Web Server issue):W465–9. 10.1093/nar/gkn180 18424797 PMC2447785

[38] Pichel M, Brengi SP, Cooper KL, Ribot EM, Al-Busaidy S, Araya P, et al Standardization and international multicenter validation of a PulseNet pulsed-field gel electrophoresis protocol for subtyping Shigella flexneri isolates. Foodborne pathogens and disease. 2012;9(5):418–24. 10.1089/fpd.2011.1067 . 22506731 PMC11359307

[39] Furuhata K, Ishizaki N, Umekawa N, Nishizima M, Fukuyama M. Pulsed-Field Gel Electrophoresis (PFGE) pattern analysis and chlorine-resistance of Legionella pneumophila isolated from hot spring water samples. Biocontrol science. 2014;19(1):33–8. . 24670616 10.4265/bio.19.33

[40] Yu J, Sun Z, Liu W, Xi X, Song Y, Xu H, et al Multilocus sequence typing of Streptococcus thermophilus from naturally fermented dairy foods in China and Mongolia. BMC microbiology. 2015;15(1):236 10.1186/s12866-015-0551-0 26497818 PMC4620635

[41] Bartlett JM, Stirling D. A short history of the polymerase chain reaction PCR protocols: Springer; 2003 p. 3–6. 10.1385/1-59259-384-4:3 12958470

[42] Wang G, Zhou F, Olman V, Li F, Xu Y. Prediction of pathogenicity islands in enterohemorrhagic Escherichia coli O157:H7 using genomic barcodes. FEBS letters. 2010;584(1):194–8. 10.1016/j.febslet.2009.11.067 . 19941858

[43] Christie PJ. Agrobacterium tumefaciens T-complex transport apparatus: a paradigm for a new family of multifunctional transporters in eubacteria. Journal of bacteriology. 1997;179(10):3085–94. 9150199 10.1128/jb.179.10.3085-3094.1997 PMC179082

[44] Flannery EL, Antczak SM, Mobley HL. Self-transmissibility of the integrative and conjugative element ICEPm1 between clinical isolates requires a functional integrase, relaxase, and type IV secretion system. Journal of bacteriology. 2011;193(16):4104–12. 10.1128/JB.05119-11 21665966 PMC3147661

[45] Zhou F, Olman V, Xu Y. Barcodes for genomes and applications. BMC bioinformatics. 2008;9:546 Epub 2008/12/19. 10.1186/1471-2105-9-546 ; PubMed Central PMCID: PMCPmc2621371. 19091119 PMC2621371

[46] Christie PJ. Type IV secretion: the Agrobacterium VirB/D4 and related conjugation systems. Biochimica et biophysica acta. 2004;1694(1–3):219–34. 10.1016/j.bbamcr.2004.02.013 . 15546668 PMC4845649

[47] Burns DL. Type IV transporters of pathogenic bacteria. Current opinion in microbiology. 2003;6(1):29–34. . 12615216 10.1016/s1369-5274(02)00006-1

[48] Xie J, Zhou F, Xu G, Mai G, Hu J, Wang G, et al Genome-wide screening of pathogenicity islands in Mycobacterium tuberculosis based on the genomic barcode visualization. Molecular biology reports. 2014;41(9):5883–9. Epub 2014/08/12. 10.1007/s11033-014-3463-4 . 25108673

[49] Zhou F, Xu Y. cBar: a computer program to distinguish plasmid-derived from chromosome-derived sequence fragments in metagenomics data. Bioinformatics. 2010;26(16):2051–2. Epub 2010/06/12. 10.1093/bioinformatics/btq299 ; PubMed Central PMCID: PMCPmc2916713. 20538725 PMC2916713

[50] Soler Bistue AJ, Birshan D, Tomaras AP, Dandekar M, Tran T, Newmark J, et al Klebsiella pneumoniae multiresistance plasmid pMET1: similarity with the Yersinia pestis plasmid pCRY and integrative conjugative elements. PloS one. 2008;3(3):e1800 10.1371/journal.pone.0001800 18350140 PMC2262945

[51] Thomson NR, Howard S, Wren BW, Holden MT, Crossman L, Challis GL, et al The complete genome sequence and comparative genome analysis of the high pathogenicity Yersinia enterocolitica strain 8081. PLoS genetics. 2006;2(12):e206 10.1371/journal.pgen.0020206 17173484 PMC1698947

