# Supplementary material for: Comparative Screening of Digestion Tract Toxic Genes in Proteus mirabilis
Source: PLoS One. 2016 Mar 24;11(3):e0151873. doi: 10.1371/journal.pone.0151873 (PMC4807080; doi:10.1371/journal.pone.0151873)

## S1 Fig

**PCR products after gel electrophoresis of the four genes in the five investigated strains of *P. mirabilis*.** (a) ureC, (b) rsmA, (c) hpmA, and (d) zapA. The lanes M and C are the 100-bp ladder and the control ddH_2_O lanes, respectively. The lanes 1 through 5 are the *P. mirabilis* strains C02011, C04010, C04013, C02034 and B02005, respectively.


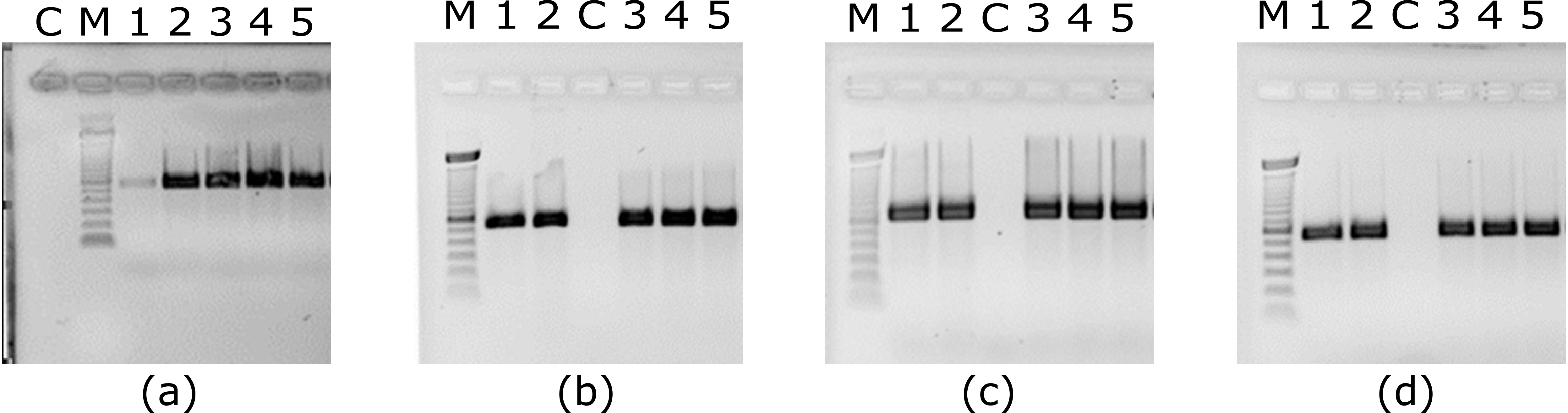

Supplement: S1 Fig — (a) ureC, (b) rsmA, (c) hpmA, and (d) zapA. The lanes M and C are the 100-bp ladder and the control ddH2O lanes, respectively. The lanes 1 through 5 are the P. mirabilis strains C02011, C04010, C04013, C02034 and B02005, respectively. (DOCX) [file pone.0151873.s001.docx]
