# Supplementary material for: Comparative Screening of Digestion Tract Toxic Genes in Proteus mirabilis
Source: PLoS One. 2016 Mar 24;11(3):e0151873. doi: 10.1371/journal.pone.0151873 (PMC4807080; doi:10.1371/journal.pone.0151873)
Supplement: S1 Table — The three candidate genomic islands are highlighted by bold font in the column “gene_id”. The annotation is provided by the BGI, using the reference databases NCBI NR, SwissProt, Trembl, COG and KEGG. (DOCX) [file pone.0151873.s002.docx]

## S1 Table

**Annotations of the 45 genes specific in P. mirabilis C02011.** The three candidate genomic islands are highlighted by bold font in the column “gene_id”. The annotation is provided by the BGI, using the reference databases NCBI NR, SwissProt, Trembl, COG and KEGG.

| **Scaffold** | **Start** | **End** | **Strand** | **gene_id** | **nr** | **swissprot** | **trembl** | **cog** | **kegg** |
| --- | --- | --- | --- | --- | --- | --- | --- | --- | --- |
| fa_scaffold3 | 465840 | 465962 | + | **C02011GL001038** | NA | NA | NA | NA | NA |
| fa_scaffold3 | 465952 | 466815 | + | **C02011GL001039** | NA | NA | NA | NA | NA |
| fa_scaffold3 | 466833 | 467849 | + | **C02011GL001040** | unnamed protein product [Xenorhabdus bovienii SS-2004] | NA | D3V746_XENBS Putative phage gene OS=Xenorhabdus bovienii (strain SS-2004) GN=XBJ1_4372 PE=4 SV=1 | NA | NA |
| fa_scaffold3 | 467846 | 468379 | + | **C02011GL001041** | unnamed protein product [Xenorhabdus bovienii SS-2004] | VPO_BPHP1 Probable capsid-scaffolding protein OS=Haemophilus phage HP1 PE=4 SV=1 | D3V747_XENBS Putative uncharacterized protein OS=Xenorhabdus bovienii (strain SS-2004) GN=XBJ1_4373 PE=4 SV=1 | NA | NA |
| fa_scaffold3 | 468467 | 468952 | + | **C02011GL001042** | unnamed protein product [Xenorhabdus nematophila ATCC 19061] | VASH_BPP4 Protein ash OS=Enterobacteria phage P4 GN=cI PE=4 SV=2 | D3VGZ2_XENNA Putative bacteriophage protein OS=Xenorhabdus nematophila (strain ATCC 19061 / DSM 3370 / LMG 1036 / NCIB 9965 / AN6) GN=XNC1_0189 PE=4 SV=1 | NA | NA |
| fa_scaffold3 | 468949 | 469179 | + | **C02011GL001043** | unnamed protein product [Xenorhabdus bovienii SS-2004] | NA | D3V749_XENBS Putative uncharacterized protein OS=Xenorhabdus bovienii (strain SS-2004) GN=XBJ1_4375 PE=4 SV=1 | NA | NA |
| fa_scaffold3 | 469224 | 471878 | + | **C02011GL001044** | unnamed protein product [Xenorhabdus bovienii SS-2004] | NA | D3V750_XENBS Putative inner membrane protein (Modular protein) OS=Xenorhabdus bovienii (strain SS-2004) GN=XBJ1_4376 PE=4 SV=1 | COG5519 Superfamily II helicase and inactivated derivatives L Replication, recombination and repair ; | K06919 K06919 -- -- Unclassified; Poorly Characterized; General function prediction only |
| fa_scaffold3 | 472166 | 472393 | + | **C02011GL001045** | unnamed protein product [Xenorhabdus bovienii SS-2004] | NA | D3V751_XENBS Putative phage gene OS=Xenorhabdus bovienii (strain SS-2004) GN=XBJ1_4377 PE=4 SV=1 | NA | NA |
| fa_scaffold3 | 472383 | 472538 | + | **C02011GL001046** | NA | NA | NA | NA | NA |
| fa_scaffold3 | 473119 | 473679 | + | **C02011GL001047** | NA | NA | NA | NA | NA |
| fa_scaffold3 | 473682 | 475016 | + | **C02011GL001048** | retron-type reverse transcriptase [Vibrio cholerae TMA 21] | NA | C2ITY1_VIBCH Retron-type reverse transcriptase OS=Vibrio cholerae TMA 21 GN=VCB_002349 PE=4 SV=1 | NA | NA |
| fa_scaffold3 | 1068445 | 1069563 | - | C02011GL001596 | hypothetical protein HMPREF9536_00816 [Escherichia coli MS 84-1] | NA | E6BSF3_ECOLX Putative uncharacterized protein OS=Escherichia coli MS 85-1 GN=HMPREF9350_05360 PE=4 SV=1 | NA | NA |
| fa_scaffold3 | 1069568 | 1070161 | - | C02011GL001597 | NA | NA | NA | NA | NA |
| fa_scaffold3 | 1535544 | 1536122 | - | C02011GL002036 | hypothetical protein PROSTU_01165 [Providencia stuartii ATCC 25827] | NA | B2PX62_PROST Putative uncharacterized protein OS=Providencia stuartii ATCC 25827 GN=PROSTU_01165 PE=4 SV=1 | NA | NA |
| fa_scaffold3 | 1536545 | 1537150 | + | C02011GL002037 | integrase core domain protein [Escherichia coli MS 182-1] | NA | D7YRA0_ECOLX Integrase core domain protein OS=Escherichia coli MS 182-1 GN=HMPREF9548_05144 PE=4 SV=1 | COG4584 Transposase and inactivated derivatives L Replication, recombination and repair ; | NA |
| fa_scaffold3 | 1538064 | 1538282 | + | C02011GL002039 | NA | NA | NA | NA | NA |
| fa_scaffold3 | 1538847 | 1539260 | - | C02011GL002041 | hypothetical protein HMPREF0693_2116 [Proteus mirabilis ATCC 29906] | NA | C2LJS6_PROMI Putative uncharacterized protein OS=Proteus mirabilis ATCC 29906 GN=HMPREF0693_2116 PE=4 SV=1 | NA | NA |
| fa_scaffold3 | 1543281 | 1543748 | - | C02011GL002049 | NA | NA | NA | NA | NA |
| fa_scaffold3 | 1543975 | 1544586 | - | C02011GL002050 | unnamed protein product [Enterobacter aerogenes KCTC 2190] | NA | G0DYZ8_ENTAE Putative uncharacterized protein OS=Enterobacter aerogenes GN=EAE_21870 PE=4 SV=1 | NA | NA |
| fa_scaffold3 | 1544614 | 1545003 | - | C02011GL002051 | hypothetical protein ESA_03903 [Cronobacter sakazakii ATCC BAA-894] | NA | A7MQ50_ENTS8 Putative uncharacterized protein OS=Enterobacter sakazakii (strain ATCC BAA-894) GN=ESA_03903 PE=4 SV=1 | NA | NA |
| fa_scaffold3 | 1809941 | 1810177 | - | **C02011GL002280** | hypothetical protein ERHG_00057 [Escherichia coli TA007] | NA | E9Y8T8_ECOLX Putative uncharacterized protein OS=Escherichia coli TA007 GN=ERHG_00057 PE=4 SV=1 | NA | NA |
| fa_scaffold3 | 1810202 | 1810420 | - | **C02011GL002281** | NA | NA | NA | NA | NA |
| fa_scaffold3 | 1810632 | 1811384 | - | **C02011GL002282** | putative phage regulatory protein [Serratia symbiotica str. Tucson] | NA | E9CLV8_9ENTR Putative phage regulatory protein OS=Serratia symbiotica str. Tucson GN=SSYM_1248 PE=4 SV=1 | NA | NA |
| fa_scaffold3 | 1811504 | 1812076 | - | **C02011GL002283** | BRO family protein [Escherichia coli MS 117-3] | NA | E9TCC1_ECOLX BRO family protein OS=Escherichia coli MS 117-3 GN=HMPREF9542_01417 PE=4 SV=1 | COG3617 Prophage antirepressor K Transcription ; | NA |
| fa_scaffold3 | 1812403 | 1813122 | + | **C02011GL002284** | NA | NA | NA | NA | NA |
| fa_scaffold3 | 1823562 | 1824128 | - | C02011GL002300 | putative phage terminase, small subunit [Yersinia enterocolitica subsp. enterocolitica 8081] | NA | A1JR11_YERE8 Putative phage terminase, small subunit OS=Yersinia enterocolitica serotype O:8 / biotype 1B (strain 8081) GN=YE2326 PE=4 SV=1 | COG3728 Phage terminase, small subunit L Replication, recombination and repair ; | K07474 xtmA phage terminase small subunit -- Unclassified; Genetic Information Processing; Replication, recombination and repair proteins |
| fa_scaffold3 | 1824196 | 1824423 | + | C02011GL002301 | NA | NA | NA | NA | NA |
| fa_scaffold3 | 1824634 | 1824879 | - | C02011GL002302 | hypothetical protein HMPREF0864_02193 [Enterobacteriaceae bacterium 9_2_54FAA] | NA | E5YI33_9ENTR Putative uncharacterized protein OS=Enterobacteriaceae bacterium 9_2_54FAA GN=HMPREF0864_02193 PE=4 SV=1 | NA | NA |
| fa_scaffold3 | 1825050 | 1825649 | - | C02011GL002303 | KilA-N domain-containing protein [Enterobacteriaceae bacterium 9_2_54FAA] | NA | E5YDF5_9ENTR KilA-N domain-containing protein OS=Enterobacteriaceae bacterium 9_2_54FAA GN=HMPREF0864_00778 PE=4 SV=1 | NA | NA |
| fa_scaffold6 | 547377 | 548129 | - | **C02011GL003316** | MobC [Klebsiella pneumoniae] | NA | B2CBE3_KLEPN MobC OS=Klebsiella pneumoniae GN=mobC PE=4 SV=1 | NA | NA |
| fa_scaffold6 | 548138 | 549889 | - | **C02011GL003317** | MobB [Klebsiella pneumoniae] | NA | B2CBE2_KLEPN MobB OS=Klebsiella pneumoniae GN=mobB PE=4 SV=1 | NA | NA |
| fa_scaffold6 | 549859 | 550503 | - | **C02011GL003318** | hypothetical protein YP_pCRY21 [Yersinia pestis biovar Microtus str. 91001] | NA | Q74YS7_YERPE Putative uncharacterized protein OS=Yersinia pestis GN=YP_pCRY21 PE=4 SV=1 | NA | NA |
| fa_scaffold6 | 550993 | 551298 | - | **C02011GL003319** | ORF15 [Klebsiella pneumoniae] | NA | B2CBE0_KLEPN ORF15 OS=Klebsiella pneumoniae PE=4 SV=1 | NA | NA |
| fa_scaffold6 | 551334 | 551708 | - | **C02011GL003320** | hypothetical protein ECED1_2271 [Escherichia coli ED1a] | NA | B7MWJ2_ECO81 Putative uncharacterized protein OS=Escherichia coli O81 (strain ED1a) GN=ECED1_2271 PE=4 SV=1 | NA | NA |
| fa_scaffold6 | 551729 | 552754 | - | **C02011GL003321** | type IV secretory pathway VirB11 component [Yersinia pestis biovar Microtus str. 91001] | VIRBB_BRUSU Type IV secretion system protein virB11 OS=Brucella suis biovar 1 (strain 1330) GN=virB11 PE=1 SV=1 | Q74YT2_YERPE Type IV secretory pathway VirB11 component OS=Yersinia pestis GN=virB11 PE=4 SV=1 | COG0630 Type IV secretory pathway, VirB11 components, and related ATPases involved in archaeal flagella biosynthesis NU Cell motility ; Intracellular trafficking, secretion, and vesicular transport ; | K03196 virB11 type IV secretion system protein VirB11 -- Environmental Information Processing; Membrane Transport; Bacterial secretion system [PATH:ko03070] Environmental Information Processing; Membrane Transport; Secretion system [BR:ko02044] |
| fa_scaffold6 | 552744 | 554012 | - | **C02011GL003322** | VirB10 [Klebsiella pneumoniae] | NA | B2CBD7_KLEPN VirB10 OS=Klebsiella pneumoniae GN=virB10 PE=4 SV=1 | NA | K03195 virB10 type IV secretion system protein VirB10 -- Environmental Information Processing; Membrane Transport; Bacterial secretion system [PATH:ko03070] Environmental Information Processing; Membrane Transport; Secretion system [BR:ko02044] |
| fa_scaffold6 | 554056 | 554961 | - | **C02011GL003323** | putative type IV secretory pathway VirB9 component [Escherichia coli UMN026] | NA | B7NBZ2_ECOLU Putative type IV secretory pathway VirB9 component OS=Escherichia coli O17:K52:H18 (strain UMN026 / ExPEC) GN=ECUMN_2293 PE=4 SV=1 | NA | K03204 virB9 type IV secretion system protein VirB9 -- Environmental Information Processing; Membrane Transport; Bacterial secretion system [PATH:ko03070] Environmental Information Processing; Membrane Transport; Secretion system [BR:ko02044] |
| fa_scaffold6 | 554961 | 555644 | - | **C02011GL003324** | type IV secretion system VirB8 component [Yersinia pestis biovar Microtus str. 91001] | NA | Q74YT5_YERPE Type IV secretion system VirB8 component OS=Yersinia pestis GN=virB8 PE=4 SV=1 | NA | K03203 virB8 type IV secretion system protein VirB8 -- Environmental Information Processing; Membrane Transport; Bacterial secretion system [PATH:ko03070] Environmental Information Processing; Membrane Transport; Secretion system [BR:ko02044] |
| fa_scaffold6 | 555866 | 556936 | - | **C02011GL003325** | hypothetical protein HMPREF1024_05167 [Klebsiella sp. 4_1_44FAA] | NA | B2CBD3_KLEPN VirB6 OS=Klebsiella pneumoniae GN=virB6 PE=4 SV=1 | NA | K03201 virB6 type IV secretion system protein VirB6 -- Environmental Information Processing; Membrane Transport; Bacterial secretion system [PATH:ko03070] Environmental Information Processing; Membrane Transport; Secretion system [BR:ko02044] |
| fa_scaffold6 | 557184 | 557885 | - | **C02011GL003326** | hypothetical protein HMPREF1024_05169 [Klebsiella sp. 4_1_44FAA] | NA | B7MW63_ECO81 Putative type IV secretory pathway VirB5 component OS=Escherichia coli O81 (strain ED1a) GN=ECED1_2264 PE=4 SV=1 | NA | K03200 virB5 type IV secretion system protein VirB5 -- Environmental Information Processing; Membrane Transport; Bacterial secretion system [PATH:ko03070] Environmental Information Processing; Membrane Transport; Secretion system [BR:ko02044] |
| fa_scaffold6 | 557903 | 560641 | - | **C02011GL003327** | hypothetical protein CKO_00896 [Citrobacter koseri ATCC BAA-895] | NA | A8AEY3_CITK8 Putative uncharacterized protein OS=Citrobacter koseri (strain ATCC BAA-895 / CDC 4225-83 / SGSC4696) GN=CKO_00896 PE=4 SV=1 | NA | K03199 virB4 type IV secretion system protein VirB4 -- Environmental Information Processing; Membrane Transport; Bacterial secretion system [PATH:ko03070] Environmental Information Processing; Membrane Transport; Secretion system [BR:ko02044] |
| fa_scaffold6 | 560654 | 560833 | - | **C02011GL003328** | hypothetical protein CKO_00897 [Citrobacter koseri ATCC BAA-895] | NA | B7NBY4_ECOLU Putative type IV secretory pathway VirB2 component OS=Escherichia coli O17:K52:H18 (strain UMN026 / ExPEC) GN=ECUMN_2285 PE=4 SV=1 | NA | K03197 virB2 type IV secretion system protein VirB2 -- Environmental Information Processing; Membrane Transport; Bacterial secretion system [PATH:ko03070] Environmental Information Processing; Membrane Transport; Secretion system [BR:ko02044] |
| fa_scaffold6 | 560947 | 561699 | - | **C02011GL003329** | putative type IV secretory pathway VirB1 component [Escherichia coli ED1a] | NA | B7MWI2_ECO81 Putative type IV secretory pathway VirB1 component OS=Escherichia coli O81 (strain ED1a) GN=ECED1_2261 PE=4 SV=1 | NA | K03194 virB1 type IV secretion system protein VirB1 -- Environmental Information Processing; Membrane Transport; Bacterial secretion system [PATH:ko03070] Environmental Information Processing; Membrane Transport; Secretion system [BR:ko02044] |
| fa_scaffold6 | 562549 | 563136 | - | **C02011GL003330** | hypothetical protein KPK_1788 [Klebsiella pneumoniae 342] | NA | B5XPP2_KLEP3 Putative uncharacterized protein OS=Klebsiella pneumoniae (strain 342) GN=KPK_1788 PE=4 SV=1 | NA | NA |
| fa_scaffold6 | 590486 | 591754 | - | C02011GL003343 | integrase family protein [Dickeya dadantii Ech703] | VINT_BPP4 Integrase OS=Enterobacteria phage P4 GN=int PE=3 SV=2 | C6CCG5_DICDC Integrase family protein OS=Dickeya dadantii (strain Ech703) GN=Dd703_3034 PE=4 SV=1 | NA | K10907 K10907 aminotransferase 2.6.1.- Unclassified; Metabolism; Amino acid metabolism |
